# Sexual harassment: The most challenging issue of medical professionalism in Japan

**DOI:** 10.1002/jgf2.186

**Published:** 2018-06-19

**Authors:** Mano Soshi, Yasuharu Tokuda

**Affiliations:** ^1^ Minami Seikyo Hospital Aichi Japan; ^2^ Muribushi Okinawa for Teaching Hospitals Okinawa Japan

Japanese society, in general, has recently supported policies against sexual harassment. Many hospitals and medical schools were required to introduce institutional committees for preventing or managing this issue. Healthcare workers, including physicians, should have had an opportunity learning appropriate and inappropriate behaviors about sexual harassment. The #MeToo movement in global social media has also influenced Japanese society. There have been formal accusations against offenders, including a journalist and a government official with strong political power, who were alleged to conduct a sexual harassment throughout Japan.^1^, ^2^

Medical professionalism has been the basis of physicians’ contract with society all over the world, and physicians in Japan are also considered to have the accountability to follow its moral principles. Thus, we previously conducted two surveys in 2005 and 2013, using a validated questionnaire (Barry Questionnaire, see Appendix 1) with six common scenarios related to professionalism.^3^ Each of six cases was related to the following issues: acceptance of gifts; conflict of interest; patient confidentiality; physician impairment; sexual harassment; and honesty in formal documentation.

The scenarios accompanied multiple choice responses, including the “best” and the “second‐best” responses.

Our results suggested that many resident physicians in Japan encounter challenges when responding to the scenarios related to professionalism.^4^, ^5^ Compared to residents in the 2005 survey, those in the 2013 survey performed better for five scenarios (gifts, conflict of interest, confidentiality, impairment, and honesty) but not for the harassment scenario. Therefore, we proposed the implementation of a validated assessment tool and the improvement of curriculum for teaching professionalism to students and residents.^6^, ^7^, ^8^

As it was unclear how Japanese staff physicians perceive these six common scenarios related to professionalism, we conducted a survey for Japanese staff physicians using the same Barry Questionnaire. Participants in our survey included physicians of primary care, general medicine, or specialty throughout Japan. They were voluntarily invited through a members‐only medical portal website (M‐Three) in April 2018. The sponsor for this community had no role in design and data analysis of the survey. The participant was involved with obtaining a point gift for the online portal. In a total of 1000 participants, there were 916 (92%) men and 84 (8%) women physicians with median 26 years of clinical experience. In all participants, 42% were physicians of internal medicine; 707 participants (71%) worked in hospitals, in which 117 (17%) worked mainly in university hospitals.

The current survey revealed the results like that for resident physicians. The most challenging was the sexual harassment scenario and only 46% provided the best or second‐best responses (Figure 1). Some (n = 42, 4.2%; 41 men and one woman) chose the response A (Do nothing, on the basis that the faculty member was simply showing his appreciation for a job well done), which seemed problematic because of under‐recognition of the harassment behavior. The next most challenging was the patient confidentiality scenario with 53%. Staff physicians were more likely to provide the best or second‐best responses to the scenarios related to conflict of interest (88%), honesty (82%), gifts (76%), and physician impairment (71%).

**Figure 1:**
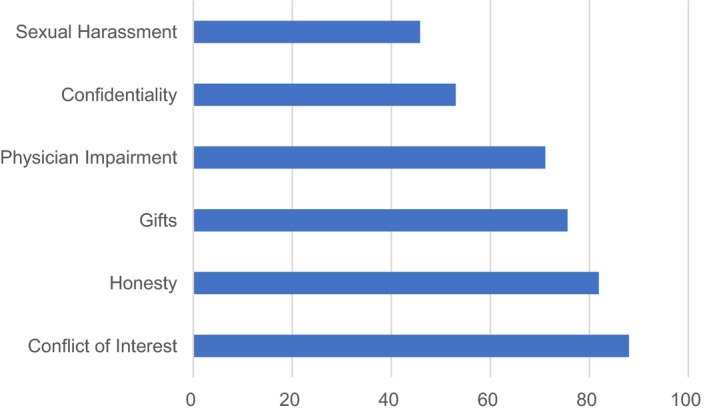
Proportion (%) of the best or second‐best responses to each scenario of medical professionalism

The current results suggest that many Japanese staff physicians were also unable to provide an acceptable response to challenges to professionalism in issue of sexual harassment. Japanese hospitals may need to widely address sexual harassment policy and establish reporting procedures and counseling for victims and witnesses.

The response B (Report the incident to the program director as an example of sexual harassment) was selected most frequently (29%) and it may reflect the difficulty in confronting or approaching an offending physician who may likely be in powerful position in any organizations. The Barry Questionnaire considered it not to be best or second‐best responses as a professional action. Now it may be the time that we should recognize this action as less appropriate and deal with it as professionals.

## CONFLICT OF INTEREST

The authors have stated explicitly that there are no conflicts of interest in connection with this article.

## APPENDIX 1

### Barry Professionalism Questionnaire

Please read the following cases. Recognizing that there may be other approaches, select the single best answer from those listed.

(1) A pharmaceutical company approaches you about a clinical research project involving your office patients. Your patients with high blood pressure will be eligible to be treated with a new medication that has just been released by the FDA. The object of the study is to evaluate risks and benefits of this medication in an unselected office population. The pharmaceutical company will pay $250 per patient for the expenses generated by the study, and 1 year's salary for a data manager and will supply the drug free of charge. Meetings to discuss the initiation of the study and follow‐up results will be held in New Orleans and Honolulu. Your spouse will be invited as the company's guest to attend these meetings since they will take you away from home.

Participating in the study would be considered appropriate professional behavior if:

A. Your patients signed an informed consent;

B. Your patients sign an informed consent and your partners approve the study;

C. An oversight committee of the hospital where you have privileges or your regional medical society approves the study;

D. None of the above.

(2) You are practicing hematology and oncology in a suburb of a large metropolitan area. Currently, you refer your patients who require radiotherapy to one or two hospitals in the city depending on where the patients live and the type of problem. A radiotherapist whose knowledge and skill you respect informs you that she will be joining a for‐profit national radiotherapy company that is thinking of locating in your area. This new company will bring both the latest equipment and up‐graded service to your community. She informs you that an excellent opportunity now exists to invest in this company and that the larger the number of investors from the area, the greater the likelihood the company will locate the unit in your community.

Which of the following statements most accurately assesses the possibility of conflict of interest regarding your investment in this company?

A. An investment will pose a conflict of interest and you should not make it.

B. Your investment will pose no conflict of interest because the new radiotherapy unit will offer superior treatment and will be available to your patients.

C. There is a possibility of conflict of interest that requires that you inform your patients of the investment.

D. Your investment will pose no conflict of interest if you avoid referring your patients to the new radiotherapy unit.

E. There will be no problem of conflict of interest for you if the investment is made by your spouse.

(3) A friend's 16‐year‐old daughter visits your office requesting birth control pills. Her family is Catholic and against birth control and premarital sex. She requests you do not discuss this with her parents. After concluding the visit, you return to your desk where you find a message to call the patient's mother. In the past you have always discussed the daughter's health and concerns openly. What will you do?

A. Call the mother back and disclose the reason for her daughter's visit.

B. Return the call and tell the patient's mother you can't discuss the matter, knowing this will look suspicious to her.

C. Return the call but be evasive when questioned about the nature of the visit.

D. Don't return the call.

(4) You are the chief of service at a hospital and a medical student informs you that she smelled alcohol on the breath of an attending physician during morning rounds on more than one occasion. This report is confirmed by another student and a resident. How do you proceed?

A. Approach the physician in question and ask if he/she has a drinking problem.

B. Talk to friend and family members of the physician to see if they suspect a drinking problem.

C. Review the physician's file and monitor him/her closely.

D. Report the physician to the Colorado Board of Medical Examiners.

(5) During your rounds with the housestaff team, a male staff member comes up to the group, places his arm around the waist of a female house officer, and thanks her for the terrific job she did she did taking care of one of his patients. You sense that the house officer is made uncomfortable by the gesture. An appropriate first response would be which of the following?

A. Do nothing, on the basis that the faculty member was simply showing his appreciation for a job well done.

B. Report the incident to the program director as an example of sexual harassment.

C. Tell your colleague, the faculty member, that you thought the gesture was inappropriate and that you were made uncomfortable by it.

D. Ask the resident if the gesture made her uncomfortable.

E. Ask the resident if there are actions she would like you take on her behalf.

(6) An established patient of yours presents with symptoms of depression. This is the second time in 3 months that the patient has visited you for these complaints. You wish to start treatment with anti‐depressant medication. As you are filling out the prescription, the patient asks you not to document the diagnosis or medication in the chart. She is concerned that her employer will find out about her diagnosis and she could potentially lose her job like a coworker did. She knows that her insurance company has access to her diagnosis. How do you proceed?

A. Inform the patient that you must document the diagnosis to provide any treatment.

B. Agree to not document the diagnosis but prescribe the medication anyway.

C. Agree to not document the diagnosis but refuse to provide the prescription.

D. Terminate your relationship with the patient because she is inhibiting your ability to provide adequate care.

E. Document an alternative diagnosis, such as fatigue, and provide the prescription.

The best responses were D for scenario 1, A for 2, B for 3, D for 4, C for 5, and A for 6. The second‐best responses were C for 1, C for 2, C for 3, A for 4, E for 5, and C for 6.
